# Rural definition of health: a systematic literature review

**DOI:** 10.1186/s12889-015-1658-9

**Published:** 2015-04-14

**Authors:** Charles Gessert, Stephen Waring, Lisa Bailey-Davis, Pat Conway, Melissa Roberts, Jeffrey VanWormer

**Affiliations:** Essentia Institute of Rural Health, 502 E 2nd St, Duluth, MN 55805 USA; Geisinger Center for Health Research, Danville, PA 17822 USA; LCF Research, Albuquerque, NM 87106 USA; Marshfield Clinic Research Foundation, Marshfield, WI 54449 USA

**Keywords:** Rural health, Patient activation, Health education, Patient-centered care

## Abstract

**Background:**

The advent of patient-centered care challenges policy makers, health care administrators, clinicians, and patient advocates to understand the factors that contribute to effective patient activation. Improved understanding of how patients think about and define their health is needed to more effectively “activate” patients, and to nurture and support patients’ efforts to improve their health. Researchers have intimated for over 25 years that rural populations approach health in a distinct fashion that may differ from their non-rural counterparts.

**Methods:**

We conducted a systematic review of the literature to assess the extent and strength of evidence for rural definition of health. Studies were eligible for inclusion if they were published in English, reported on original research and presented findings or commentary relevant to rural definition of health, were published over the last 40 years, and were based on observations of rural U.S., Canadian, or Australian populations. Two reviewers were assigned to each selected article and blinded to the other reviewer’s comments. For discordant reviews, a third blinded review was performed.

**Results:**

Of the 125 published articles identified from the literature, 34 included commentary or findings relevant to a rural definition of health. Of these studies, 6 included an urban comparison group. Few studies compared rural and urban definitions of health directly. Findings relevant to rural definition of health covered a broad range; however, good health was commonly characterized as being able to work, reciprocate in social relationships, and maintain independence.

This review largely confirmed many general characteristics on rural views of health, but also documented the extensive methodological limitations, both in terms of quantity and quality, of studies that empirically compare rural vs. urban samples. Most notably, the evidence base in this area is weakened by the frequent absence of parallel comparison groups and standardized assessment tools.

**Conclusions:**

To engage and activate rural patients in their own healthcare, a better understanding of the health beliefs in rural populations is needed. This review suggests that rural residents may indeed hold distinct views on how to define health, but more rigorous studies are needed to confirm these findings.

**Electronic supplementary material:**

The online version of this article (doi:10.1186/s12889-015-1658-9) contains supplementary material, which is available to authorized users.

## Background

The passage of the Affordable Care Act (ACA) in 2010 heralded a significant shift in American health care, with increased emphasis on population health, disease prevention, and cost containment. These new areas of emphasis are prominent in several initiatives that have emerged in the wake of the ACA, including Accountable Care Organizations (ACOs), which tie provider reimbursements to measures of quality of care and reductions in the total cost of care for assigned populations of patients. The Patient Centered Outcomes Research Institute (PCORI) also emerged, charged with examining the relative health outcomes, clinical effectiveness, and appropriateness of medical treatments. Underlying these initiatives is a renewed emphasis on patient engagement in health care and, more specifically, on patient activation.

While *patient engagement* and *patient activation* are often used interchangeably, *patient engagement* denotes the broad involvement of patients and caregivers in all aspects of health care is based on the principle of shared responsibility [1]. *Patient activation*, a component of patient engagement, emphasizes patients’ willingness and ability to take independent actions to manage their health and care [2]. Patient activation has been found to be associated with better health outcomes, better health care experiences, and reduced probability of adverse markers such as emergency department use, obesity, and smoking [2,3]. Health care systems are encouraging primary care providers to practice patient-centered care, employing strategies that engage with and activate patients. This approach is grounded in the perspective that care should focus on patients and finding a common ground between patients and clinicians when choosing preventive and treatment care pathways.

This focus on patient-centered care challenges policy makers, health care administrators, clinicians, and patient advocates to understand the factors that contribute to effective patient activation. Individuals define “health” on the basis of their personal health-related beliefs, values and knowledge. Improved understanding of how patients think about and define their health is needed to more effectively “activate” patients, and to nurture and support patients’ efforts to improve their health.

For more than 25 years, researchers have suggested that rural populations may have a distinct view of health that differs from other non-rural populations. Seminal research in 1987 by Weinert and Long reported that rural people predominantly associated health with “the ability to work” [4], but were less likely to regard cosmetic, comfort, or life-prolonging aspects of health as important. In subsequent work, Weinert and Burman concluded that the rural “function-based definition of health” may contribute to delays in seeking health care, even in the face of grave illness [5]. In a study of the health beliefs of rural elders, Davis et al. found that subjects described health in terms of autonomy and self-reliance; they feared loss of health primarily because it could lead to “being a burden to others” [6].

These early studies indicate the need for a richer understanding of rural “frames” for health, but they lacked a direct comparison to health views from non-rural counterparts. If rural and non-rural populations indeed commonly think about or define their health differently, then efforts to engage such populations in promoting and preserving health must be better informed, particularly as health care providers increasingly focus on patient-centered care. The purpose of this study was to systematically review and critique the extent and strength of the published literature regarding how persons living in rural areas define health. In addition, we sought comparisons between rural and urban concepts of health. We were interested in findings that could guide improved patient engagement and patient activation in rural communities of the United States and similar industrialized countries. We specifically examined health values and beliefs as constructs rather than knowledge *per se*, as knowledge generation is better understood as a continuous process influenced by values, beliefs, motivation, skills, and context [7,8].

## Methods

We conducted a systematic review of the literature to assess the current scientific understanding of rural definitions of health [9]. The online databases PubMed, CINAHL, PsycINFO, AnthroSource, and Sociological Abstracts were searched, followed by a manual search of the reference sections of studies identified through the online database search. Key search terms that were used were “rural population, “attitude to health,” “health behavior,” “health promotion,” “health belief,” and “health values.” Studies were eligible for inclusion if they were published in English, reported on original research, presented findings or commentary relevant to rural definitions of health, were published over the last 40 years (01/01/1972-03/31/14), and were based on observations of rural U.S., Canadian, or Australian populations. These countries were selected because of their large rural populations, including remote/frontier communities, and their access to Western health care. No restrictions were placed on sample size, research design, or length of follow-up.

For data extraction and synthesis, two reviewers were assigned to each article and blinded to the other reviewer’s comments. For discordant reviews, a third blinded review was performed. Articles were reviewed for content, methodology and rigor, with information collected on study design, characteristics of the study population, whether articles related to rural definitions of health, the definition of rural, and whether there was a comparison group (e.g., rural vs. urban). All information was captured in a Microsoft Excel spreadsheet for summarizing and comparisons. Further details on the review process are provided in Additional file 1.

The review process identified 34 articles as having fulfilled the criterion of assessing how rural residents define health. These articles were assigned an evidence grade of A, B, or C depending on methodological quality and supporting evidence of the conclusions, based on a previously used adaptation of the American Diabetes Association's (ADA) evidence grading system [10,11].

Because this was a retrospective review of data from previous published studies, no patient informed consent procedures were applicable, and the study was exempt from review by the Essentia Health Institutional Review Board.

## Results

**Figure 1:**
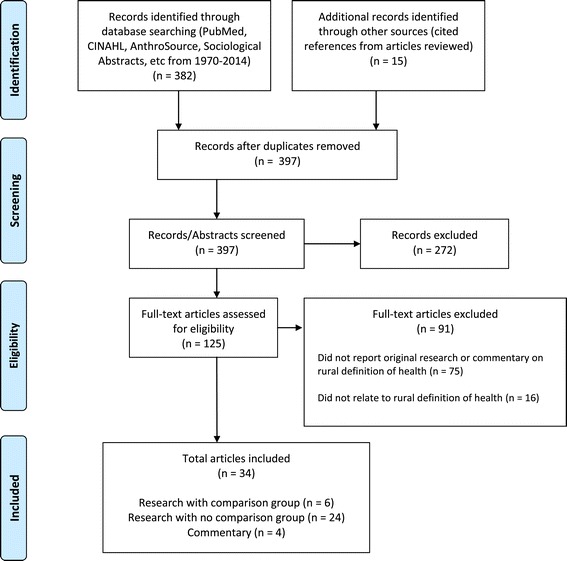
PRISMA diagram of literature review process.

Three hundred and eighty two articles were identified using the study’s search terms; an additional fifteen articles were identified as cited references during the review process. From these, 125 articles were selected for initial review by the lead author. Ninety-one articles were excluded because they did not report on original research or were outside of the scope of the current inquiry. Thirty-four were utilized for this review; 4 were commentaries about a rural definition of health [4,12-14] and 30 contained findings relevant to a rural definition of health (see Figure 1). Of the latter 30 studies, 6 [15-20] included an urban comparison group (see Table 1). The remaining 24 articles [6,21-43] did not include a comparison group. Few studies compared rural and urban definitions of health directly.

**Table 1:** **Published research relevant to rural definition of health: with comparison groups (N = 6) and without comparison groups (N = 24)**

**Author**	**Study design**	**Study population**	**Findings**	**Quotes (Participants)**	**Quotes (Investigators)**
*Articles reporting definitions of health by ural residents compared to urban residents*					
Gessert, 2006 [15]	Qualitative – focus groups	Minnesota	• Rural respondents tended to characterize death as natural; death was seen as neutral or beneficent.	Rural respondents: “When the body starts shutting down there isn’t a need for food.” “Sometimes they just wear out. We [are] just like an old car.”	“Resistance to the approach of death was more characteristic of urban respondents, some of whom insisted upon aggressive medical care in advanced dementia.”
		• 8 focus groups in rural and urban nursing homes	• Urban respondents described “fighting” for their relatives’ survival; death was not accepted by many.	Urban respondents: “You have to fight for their rights … to live.” “I think things should be done as if she were just 20 years younger.”	
		• 38 family members (average age 62) of elders (age 65+) with advanced cognitive impairment	• Resistance to approach of death more characteristic of urban respondents; some insisted upon aggressive medical care in advanced dementia.		
Harju, 2006 [16]	Telephone survey	North Carolina	• Fear of hospitals was associated with effective compliance for rural residents and good health habits for urbanites.	(No quotes from participants)	“Affordability concerns of rural residents were associated with both noncompliance and irregular health habits.”
		• Rural (N = 586, average age 46) and Urban (N = 433, average age 44) respondents based on random digit dialing	• Mistrust of doctors predicted low adherence for both groups and was also associated with poorer health habits for urbanites.		
Hoyt, 1997 [17]	Longitudinal survey of health and service use	Iowa	• There were significant differences [by size of place] for financial distress, with the greatest levels of stress reported by farm residents, followed by persons living in rural villages and small towns.	(No quotes from participants)	“Persons living in the most rural environments were more likely to hold stigmatized attitudes toward mental health care and these views were strongly predictive of willingness to seek care.”
		• 1487 adults completing the full battery of mental health questions in both waves of data collection (separated by >1 year)	• Persons in rural areas expressed significantly higher levels of stigma [toward seeking mental health care] than residents of population centers.		
King, 2006 [18]	Qualitative - semi-structured interviews	Alberta, Canada	• Rural-living participants believed that a ‘work hard, eat hard’ ethic kept them healthy despite stressors related to the nature of their work and living in a rural environment.	Rural respondents: “See, the family depends on me. No matter if I work 24 hours a day or 12 hours a day, I have to be there.”	“…gender and culture (associated with place of residence) influence people’s ability to meet the challenge of managing [coronary artery disease] risk.”
		• 42 urban- and rural-living Euro-Celtic men and women (average age 63)	• Participants persevered through CAD symptoms until they could no longer undertake their daily activities related to maintaining the household or farm.	“…when a farmer or a rancher phones you … you see them right now because they only let you know if it … is really, really serious.”	
Rakauskas, 2009 [19]	Survey, mailed to a stratified sample of adult drivers	Minnesota	• Rural drivers participate in risky driving behaviors more than their urban counterparts and tend to regard the risks of the behaviors, such as not wearing seat belts, as being lower.	(No quotes from participants)	“…traffic safety policy to improve seatbelt compliance in rural areas should focus on increasing the perception of danger associated with not using a seatbelt while driving.”
		• 1399 valid survey responses from three age groups (age 18-26, 30-50, and 65+) of MN licensed drivers.	• Rural drivers had significantly lower confidence in the utility of safety interventions such as traffic enforcement and highway engineering.		
Thorson, 1992 [20]	Survey research	Nebraska	• Rural elders were less inclined to turn to health care providers for issues that they considered non-urgent.	(No quotes from participants)	“Those in the rural group expressed attitudes of independence and self-reliance, values consistent with concepts of pioneer virtues and responsibility.”
		• 396 people (aged 65 or older) in Omaha (urban, n = 196) and Sand Hills (rural, n = 200)	• Rural residents emphasized independence items such as waiting long enough to get over illness and avoiding doctors and hospitals.		
*Articles reporting definitions of health by rural residents without comparisons to non-urban residents*					
Arcury, 2001 [21]	3-year ethnographic study; qualitative research (focus: health maintenance)	North Carolina	• Rural residents identified 7 salient health maintenance domains: eating right, drinking water, taking exercise, staying busy, being with people, trusting in God and participating in church, and taking care of yourself.	“[To be healthy] get involved in church and community activities.”	“Elders in these rural communities hold a definition of health that overlaps with, but is not synonymous with a biomedical model. These elders concept of health seamlessly integrates physical, mental, spiritual, and social aspects of health,”
		• 145 interviews with residents of 2 rural NC counties (aged 70+)		“And go to church… keep up your activity in the church, your singing, your praying and your studying the Bible…”	
Arcury, 2005 [22]	3-year ethnographic study; qualitative research (focus: complementary & alternative medicine)	North Carolina	• CAM therapies are widely used but are largely limited to folk and home remedies and vitamin and mineral supplements.	“I had high blood pressure. … The nurse … told me she had been to [a university medical center] and somebody had advised her to mix honey and vinegar for blood pressure. So for years I took honey and vinegar.”	“The CAM used by these older adults is clearly ‘complementary’ rather than ‘alternative.’ CAM remedies are an integral component of their health self-management strategies, integrated into rather than replacing conventional care.”
		• 145 interviews with residents of 2 rural NC counties (aged 70+)	• [Rural elders’] discussions of CAM therapies … indicated that these therapies were integrated into their health behaviors and beliefs.		
Averill, 2002 [23]	Qualitative research: ethnographic interviews and participant observation	New Mexico (SW)	• For elders retired from mining and/or ranching, health consisted primarily of… remaining autonomous (living on their own in their own homes, preparing their own meals).	“I just do without a lot of things to buy my medicine. As long as I can get both food and medicine, nothing else matters. But sometimes I skip a dose or cut pills in half to make it last.”	“It is important for nurses to expand their knowledge and understanding of issues affecting elder care in the rural communities, the elders’ definitions of health, and the economics of care delivery.”
		• 22 interviews; 16 rural elders (65+ years)	• Definitions of health … encompassed … avoidance of contact with the health care system …remaining independent.	“I don’t like to call ‘em [health care providers] because they’ll just say, ‘well, you’re 85, what do you expect to happen?’”	
Bilinski, 2010 [24]	Mixed methods research using questionnaire and Body Mass Index scores	Saskatchewan, Canada	• Cultural symbols of rurality such as “wide open spaces” and the sense of “safety” and “freedom” were described and explained as contributing to health and healthy behaviors by the children and their parents.	“Healthy means being active, and having fun, and being happy.”	“…. To this group of children the meaning of being healthy was an integration of three overarching themes: Knowing Stuff, Having a Working Body, and Feeling Happy.”
		• 51 (of 99) rural students (grades 1-7) completed questionnaire and BMI; interviewed subset of 20 (age 9-12 years)		“If you don’t feel good about yourself you really have no reason to do either of the others.”	
Brown, 1990 [25]	Qualitative; interviews	Rural Nebraska	• Ideas about independence and connection were more significant to the definitions of and responses to health and illness than was the actual diagnosis of disease. According to local beliefs, one needs to be engaged with others in order to stay healthy.	“It will kill some of them if they have to be dependent on somebody else.”	“The significant cultural measure of one’s health in this town was the ability to function independently.”
		• 45 interviews with 29 selected adult respondents (more than 50% of respondents were 60+ years)		“Oh, I know I don’t go to a doctor like I probably should for a checkup … but I always feel as long as I feel half way good - why I guess it’s like a piece of machinery, as long as it’s running good, why fool with it?”	
Coyne, 2006 [26]	Qualitative research; focus groups	West Virginia	• Some participants reported that seeking help from a medical institution or provider was regarded as a last resort among people in the region.	“I believe in prayer, but I believe [God] gave doctors knowledge, also.”	“Participants stated that people in the region regard disease and accidents, like other hardships, as always a part of their lives.” “…health beliefs are strongly related to religious beliefs and practices among people in southern West Virginia.”
		• 10 focus groups (5 men, 5 women) of 61 long-time adult residents (age 35+) in rural southern WV	Religious beliefs and faith in God were important resources when facing sickness and seeking healing. For some, divine help for healing seemed to be enough.	“We have strong beliefs, and it’s not beliefs you just pick up. It’s beliefs that have been passed down from generation to generation.”	
Craig, 1994 [27]	Qualitative research; ethnographic design	Western plains US	Health was regarded as the ability to remain independent and not be a burden to anyone. Hardcore independents would not accept help from anyone.	“I wish I wasn’t so damned independent … I could get more of what I need, but it just goes against my grain.”	“The definition of health as the ability to remain active, the importance of independence, and the insular nature of communities are frequently mentioned in the literature, whereas the value of community participation is less well documented.”
		• Interviews of 104 people in a farming community; 59 with older residents (65+ years), plus community leaders and care providers	• The participants described reciprocal relationships between the community and older residents, to the benefit of both.		
Davis, 1991 [6]	Qualitative research; interviews	Rural Alabama	• To determine whether they considered themselves healthy or no longer healthy, subjects tended to rely on how they felt. They assumed that they were healthy if they felt good, whereas they knew they were no longer healthy when they felt bad.	“I know I’m healthy when I feel good. I get up and I’m not tired and I don’t have a headache.”	“The clients described a need to experience themselves as competent and self-determining. Despite multiple health problems and treatments, the subjects tended to rely first on their own appraisal and resources to manage health problems.”
		• Interviews and data collection from 31 participants ages 65-94 (n = 25 women)		“My greatest fears are that I’ll have a stroke and be a burden to my family.”	
Deskins, 2006 [28]	Qualitative research; individual and focus group interviews	Rural West Virginia	Barriers to participating in cholesterol screening programs were identified: in addition to lack of knowledge, concerns about the outcomes of testing and concerns about needles, traditional cultural beliefs were identified as barriers.	“People basically don’t come running to the doctor for minor issues and in some instances they don’t necessarily go to the doctor for major issues. … I know a lot of parents who are not open to suggestions or wanting to try new things …”	“…there are environmental, financial, and attitudinal barriers to participation in health screenings for West Virginians … denial and fatalism may also function as barriers to cholesterol screenings.”
		• Interviews with 14 community leaders, 36 parents, and 92 fifth-graders	• These beliefs included resistance to a preventive approach to health, resistance to new people and ideas, using denial as a coping strategy, and having a fatalistic view toward health.	“… you know, I’m going to die anyway. I might as well enjoy what I’m doing.”	
Freydberg, 2010 [29]	Qualitative research; interviews	Alberta, Canada	• Work and place were seen to be indivisible with work seen as integral to life in the rural setting.	“It’s not the painfulness that bothers me, it’s the unableness.”	“To be able to work was to ‘be healthy’ in the rural setting irrespective of any symptoms or underlying illness.”
		• Interviews of 42 rural elders (mean age 76) and 30 caregivers	• Accounts of heart failure and self-care were framed around narratives of work, its benefits, and the threats and disruptions made to work by heart failure.	“I don’t want to die .. I’ve still got work to do, what the hell…”	“[Work] was reported to be pleasurable and vital to a meaningful existence in the rural places.”
				“I don’t want to go [to community events] if I can’t do my share of help … I don’t like just sitting on my butt and letting everyone else to do it.”	
Goins, 2011 [30]	Qualitative research; focus groups and brief surveys	West Virginia	• These participants described clean living as one of the keys to health, and endorsed a conventional Christian way of life (i.e., reading the Bible, praying, attending church).	“…the definition of health to me is to be active.”	“…compared to more urban adults, our study participants may place greater value on functional independence due to a greater reliance on their ability to chop wood or tend to a garden.”
		• 13 focus groups and surveys (101 rural participants age 61+; average age 75 years)	• These older adults assigned a high value to health because health enabled them to remain active and to fulfill social roles.	“Keeping clean and keeping yourself busy and no idle time because idle time is in the hands of the devil…”	“The meaning of health for rural elders transcends the physical/behavioral dimensions [and includes] psychosocial and spiritual well-being.”
				“I think health is a state of mind…if you think you are healthy and you feel healthy then you are healthy.”	
Hinck, 2004 [31]	Qualitative research; interviews	Midwest US	Participants placed a high value on remaining in their own homes and remaining independent. They were creative in changing their environment and everyday practices and patterns to be able to complete most desired activities.	“I’ve worked it out. I really don’t stand up and walk up steps. I kind of crawl up. I put my hands on a couple of steps up. … You can learn to do something if you have to. I couldn’t go up stairs in public.”	“The ability to walk and other activities that were necessary for them to take care of themselves were of primary importance to participants.”
		• 59 interviews of 19 “oldest-old” rural adults (85-98 years)	• These elders viewed themselves as having strong reciprocal social networks.		
Humphreys, 2006 [32]	Mixed methods; health diary and interviews	Rural Australia	• Rural families encountered some health problem in 1 out of 2 weeks.	(No quotes from participants)	“… there was abundant evidence from the health diaries to suggest that mental-health issues remain something of a ‘sleeping giant’ in rural areas and certainly one of the most significant health problems characterizing rural families.”
		• Interviews and 4-month diary from 112 families with preschool or primary school age children	• Health care services were accessed for a wide range of illnesses.		“…notable …is the high proportion who consider the health problem not serious enough to seek assistance, even though it affected their activities.”
			• A high proportion of families experienced health problems that were sufficiently serious to affect function. Stress was widespread and impacted all members of families.		
Lee, 1993 [33]	Survey of farmers and ranchers	Montana	• The survey found that the farmers and ranchers tended to reject “the sick role.” This may be consistent with other findings that rural people experience fewer days in bed and less restricted activity and work loss, and may imply a partial reason for delays in seeking treatment for acute and chronic illnesses.	(No quotes from participants)	“In any culture, health behaviors are the result of values learned during childhood. In farming and ranching environments, children grow up in a culture that values productivity, industriousness, role performance, and independence.”
		• Survey of 162 farmers and ranchers			
Morgan, 2009 [34]	Qualitative research; focus groups	Wyoming	• Four themes emerged from the focus group data: “cowboy up” (minor health concerns should not interfere with chores and work); access to health care providers and quality care is a concern; personal and family knowledge of how to manage minor health concerns is valued; and the community is family, providing advice and care.	“You’ve got cows to feed and stuff like that. So you’ve got to ‘cowboy up’ and continue doing what you have to do.”	“…rural families may not seek treatment at the first sign of symptoms.”
		• Focus groups with 42 individuals from five rural communities (age 23-89; average age 53 years)		“We are kind of a tough lot … this community, and I think when you talk about respiratory, upper respiratory infections, most everybody ‘foofoos’ it off and takes care of themselves.”	“Mothers in our study were adamant regarding knowledge that they had regarding the symptoms and care of their family members, and in particular, the care of family members with special concerns.”
Roberto, 1992 [35]	Qualitative research; interviews	Colorado	• The rural elderly identified several factors that influenced their acceptance of health care services, including self-perception (active minds but bodies that were less cooperative); fear of dependency; financial issues; and maintaining dignity.	“In rural areas, most seniors have the attitude of, ‘if it ain’t broke, don’t fix it.”	“Nurses and other health care providers working with the rural elderly need to be sensitive to the perspective of rural older adults, which is often characterized by a lifestyle of simplicity and frugality.”
		• Interviews with 32 rural providers & 28 rural seniors (age 62-94)		“I know one old man who is 90 years old and he feeds 50 head of cattle every morning on his ranch. When he comes into my office, he can barely walk down the hallway … But he has a sense of purpose on his ranch and this keeps him going.”	
Running, 1998 [36]	Phenomeno-logical; inductive, descriptive research	NW Plains, US	The participants emphasized the value of hard work, acceptance of life’s uncertain challenges, acceptance of health and health problems, faith in God, and the importance of independence and determination.	“If something happens, it just happens, and you have to take things as they come.”	“While independence and self-care are very important qualities for the participants, their lives were so interdependent in the early years on the prairie that the stories all reflect the same underlying grounding theme.”
		• Interviews with 9 rural homesteaders (age 80-97)	• The subtheme of “acceptance” encompassed strong currents of stoicism and fatalism.	“…health I think is being able to get around to do things that you like to do and do for yourself and not have to rely on somebody else.”	
Sellers, 1999 [37]	Qualitative research; interviews	Iowa	• Rural men take health for granted and only become concerned with health when it interferes with their work and other responsibilities. They seek health care “only when the problem becomes so intense that it interferes with assumption of their roles and responsibilities.”	“Illness means you cannot go out the door to work.”	“All key informants equated health with being able to work and meet responsibilities.”
		• Interviews with 7 rural “key” male informants (age 25-49) & 12 others		“Office nurses understand how busy we [farmers] are and if it is May and you’re in the fields and come in for something, you must be near death.”	“…rural men take health for granted and only become concerned with health when it interferes with their work and other responsibilities.” …
					“…rural men seek health care only when absolutely necessary.”
Slusher, 2010 [38]	Mixed methods; interviews	Appalachian states, US	• The participants provided a highly functional definition of health, including being able to get out of bed, having energy, able to participate in activities, able to care for family and home, and able to give service to others. Health was also associated with feeling good, belief in God, feeling no pain, and never having to see a doctor.	(No quotes from participants)	“Values characteristic of the Appalachian culture include emphasis on religion, importance of family, independence, individualism, self-care, sense of place, and love of place.”
		• Interviews with 129 women from 7 states (age 19 to 101)			
Stanford, 1991[39]	Interviews; survey	Central Minnesota	• Most patients identified themselves as taking responsibility for their own health. Seventy-six percent of patients who were “careful to live a healthy lifestyle” agreed that doctors should have educational programs on healthy lifestyles available for patients.	(No quotes from participants)	“…this rural patient group had very strong interests in both primary and secondary preventive medical services.”
		• Questionnaires returned by 270 patients (age 14-90 years; average age 40 years) and 8 physicians in a rural clinic.			“Patients’ interest in physician involvement in preventive medical care … decreased significantly with increasing age, male gender, and lower levels of education.”
Tessaro, 2005 [40]	Qualitative research; focus groups	West Virginia	• In a resource-poor area like West Virginia, people adopt creative strategies for coping with chronic illness like diabetes. They may lack access to appropriate care and have limited access to [diabetes education, providers with knowledge to educate patients about diabetes management, exercise facilities and even appropriate foods for diabetes care.	“There is lots of things I have go wrong that I need to tell the doctor. But I know that I can’t go out here and pay for all these tests, so I will keep it to myself. I don’t even tell him because I know he’s going to want extensive blood work …”	“What is medically labeled as non-adherent behavior … is often a common-sense adaptation for the patient from within his or her belief framework, cultural context, and outside influences, such as financial constraints, limited knowledge, and lack of availability of appropriate medical care …”
		• 101 rural adults in 13 focus groups (average age 59)			
Thomlinson, 2004 [41]	Ethnographic study; interviews	Calgary, Manitoba	• Although a number of participants identified living with chronic illnesses, they considered themselves to be healthy; they had adapted to their illnesses. Being healthy included being able to do what they wanted to do, to cope, to enjoy themselves, to not be bored and to feel that they were productive citizens.	(No quotes from participants)	“Being healthy was described as involving the physical, mental, social, and spiritual aspects of a person. … Those health professionals who truly listen to their patients and view them as whole persons rather than disease entities are highly valued.”
		• Interviews of 29 from small towns near Calgary (age 21-84 years), 26 from northern Manitoba (age 21-76 years)			
Walker, 1994 [42]	Multi-phase project	West Virginia	• Forty-six percent of telephone survey responders agreed that “there is nothing I can do to prevent cancer.” Sixty-four percent … agreed with the statement “I would not change my habits to avoid getting cancer.” Thirty-eight percent … agreed … “I would rather not know if I have cancer.”	(No quotes from participants)	“The barriers to prevention identified by the project committee were inadequate knowledge and sources of information, physical and economic access to care, and attitudes and beliefs regarding cancer.”
		• 282 telephone surveys and (adults: 55% over the age of 45 years); 5 focus groups in rural WV			
Wathen, 2007 [43]	Qualitative research; interviews	Ontario	• Many of the women in this study exhibited characteristics of self-reliance and stoicism, sometimes in the extreme. The findings raised the question of how much self-care is reasonable. The promise of increased access to health information through the internet should be examined cautiously, as it may be of limited value to some rural populations.	[one respondent] “… told the interviewer that after breaking her arm, she did not visit the emergency service at the hospital, because she ‘didn’t want to bother the doctor or tie up the emergency … no reason to make a big drama about it’.”	“A number of women told the interviewer they would not use hospital emergency or nurse telephone triage services, even in response to the acute medical scenario, but would instead self-medicate, rely on information in home remedy books, and/or wait until morning to call their family doctor.”
		• Interviews of 40 rural women (age 20-82)			

The 6 studies that compared findings from both rural and urban populations were of primary interest. Findings relevant to a rural definition of health covered a broad range; however, good health was commonly characterized as being able to work, reciprocate in social relationships, and maintain independence (see Table 1). In a focus group study by Gessert et al., rural responders were more likely to express greater willingness to accept ill health and even death as natural phenomena, whereas urban residents expressed stronger aversion to death and greater insistence on aggressive end of life care [15]. In a study to examine factors influencing individual capacity to manage coronary artery disease risk, both gender and culture (rural vs urban) were identified [18]. Rural residents expressed belief that a “work hard, eat hard” attitude kept them healthy despite the stress of their work and living in a rural environment. Additionally, rural residents would only seek a physician’s help if physical functioning was severely impaired [18]. Rural vs. urban differences were also evident in driving behavior, with rural residents more likely to participate in risky behaviors and less likely to have confidence in the utility of safety interventions [16]. Another study found that persons living in the most remote environments were more likely to hold highly stigmatized attitudes toward mental health care and these views were strongly predictive of willingness to actually seek care [17].

Comments from participants in several of the reviewed studies (see Table 1) centered on three traits that influenced their definition of health: independence, stoicism, and fatalism. Thorson et al. found that rural elders were less likely than urban elders to turn to health care providers for issues they considered non-urgent, regardless of how long a particular symptom had been present [20]. Hoyt et al. concluded that the agrarian ideology of self-reliance and rugged independence, coupled with a lessened sense of confidentiality and increased pressure to conform due to the smaller, more intimate nature of smaller rural environments, was not conducive to seeking mental health care, particularly for males [17]. Attitudes of rural and urban residents toward seeking medical care were similar in the Harju et al. study [16], but were somewhat incongruent with self-reported care seeking behaviors. Fear of hospitals was associated with medical adherence in rural residents and good health habits in urbanites [16].

Original research articles that did not include a comparison group (n = 29) also revealed influential themes among rural residents’ definitions of health: autonomy, avoiding medical care, and spiritual health. Rural elders participating in a study in Alberta (Canada) reported that ability to work and ability to function, irrespective of symptoms or underlying illness, was their definition of “health” [29]. In a focus group study of individuals from rural communities in Wyoming, “cowboy up to continue doing what you have to do” was a prevailing theme in responses pertaining to how participants viewed health [34]. Arcury et al., reporting from interviews of elderly residents in two rural communities in North Carolina, concluded that the residents’ definition of health integrated physical, mental, spiritual, and social aspects of health [21]. Another study of rural elderly in New Mexico reported that the common definition of health consisted of remaining autonomous and independent, avoiding contact with the health care system [23]. Lastly, from a study that included interviews of rural health providers in Colorado, one provider’s perspective, based on a 90 year old patient still engaged in ranching, was that work at any age gave patients a sense of purpose that kept them going regardless of the physical challenges of getting around [35].

## Discussion

This review assessed the extent and strength of evidence regarding how rural people in the United States, Canada and Australia view health differently than their urban counterparts. The overarching objective of this review was to better understand rural definitions of health and how they might be applied in health education messaging and patient engagement/ activation strategies related to disease prevention and treatment. This review largely confirmed many general characteristics previously observed on rural views of health, but also documented the extensive methodological limitations of studies that empirically compared rural vs. urban samples. The evidence in this area is particularly weakened by the routine absence of parallel comparison groups and standardized assessment tools, among other limitations.

Despite these limitations, several consistent characteristics of a rural definition of health were identified. Rural populations tend to emphasize functional aspects of health, especially the preservation of the ability to work and to fulfill (traditional) social roles. Rural people tend to frame health in terms of independence and self-sufficiency, and to accept ill health with higher degrees of stoicism and seemingly more fatalism. If more rigorous future studies can confirm these findings in rural populations, health education and patient engagement/activation programs can be better structured in ways that capitalize on the strong underlying motivations to preserve independence through good health practices. Our findings suggest that rural populations might be more responsive to health messages that emphasize physical function, independence, self-sufficiency, and the ability to reciprocate in social roles and perceived obligations.

Projects designed to improve the health of rural populations face a number of challenges. At a macro level, rural settings are not homogenous in terms of culture, economic hardship, or sense of history/community. Accordingly, findings from one rural community or similar group of rural communities may not be applicable to other rural communities or regions. Much of the previous research on rural health reported findings from primarily agrarian samples, which is an increasingly small subset of rural settings and not necessarily similar to other rural areas that rely heavily on manufacturing, forestry, or subsistence occupations. This distinction has become more pronounced in recent years with the growth of rural recreation and retirement communities, as well as other rural environments where the agrarian economy or culture has limited influence.

Individual characteristics are also important in rural health attitudes and beliefs. Several investigators reported that religious or spiritual health was an integral part of the definition of health in the rural communities studied. Socioeconomic status is recognized as a key factor in health attitudes and practices, yet few studies in the current review controlled for the socioeconomic status of rural participants. Age and length of time in the community may also be important because some of the most distinctive rural definitions of health were held by older residents (particularly those who had a life-long history of rural residence). The current review also suggests that some work histories such as lifelong farming or ranching may be associated with the more distinct views of health framed by physical function and capacity to work. A better understanding of rural attitudes and beliefs is needed to engage and activate rural residents in managing their health and care. Thus, further study of how rural residents define health will contribute to the implementation of patient-centered care in rural communities.

This study was limited principally by its focus on industrialized Western countries. Additional research is needed both to examine rural concepts of health in a wider range of settings, especially in the developing world. This study was also limited by the paucity of rigorous studies that compared rural and urban perceptions of health directly. This is a rich arena for future research.

## Conclusions

There is increasing interest in engaging and activating patients in their own healthcare. To do so effectively in rural areas, a better understanding of the health beliefs in rural populations is needed. This review suggests that rural residents may indeed define health in their own way (e.g., functional independence). However, a formal assessment of the risk of bias was not performed in this paper because the vast majority of studies were qualitative and did not include direct comparisons between rural vs. non-rural samples. As such, selection bias remains an overshadowing concern in this collective body of literature, highlighting the need for more rigorous studies to confirm our findings. Research on rural definitions of health is further complicated by continuously changing rural lifestyles and landscapes as demographics and economic emphases shift. Despite such challenges, however, further research on rural health beliefs and attitudes is critical as American healthcare reform legislation calls for broader, systems-based strategies to improve the public’s health. To better engage and activate rural patients in their own healthcare, a better understanding of the health beliefs of targeted rural populations is needed.

## Additional file

Additional file 1:
**Literature search and review methodology.**

## Abbreviations

ACA: Affordable Care Act
ACO: Accountable Care Organization
PCORI: Patient Centered Outcomes Research Institute
SES: Socioeconomic status
