# Supplementary material for: Rural definition of health: a systematic literature review
Source: BMC Public Health. 2015 Apr 14;15:378. doi: 10.1186/s12889-015-1658-9 (PMC4406172; doi:10.1186/s12889-015-1658-9)
Supplement: Additional file 1: — Literature search and review methodology. [file 12889_2015_1658_MOESM1_ESM.pdf]

APPENDIX A – Literature search and review methodology

This appendix provides further details on the methods utilized for the systematic review. The PRISMA checklist (http://www.prisma-statement.org/statement.htm) was utilized as a reporting guideline.

Search strategy: Initial searches were conducted on July 13, 2012, and August 15, 2012, and were updated on January 23, 2014. In each case, Boolean logic was used. Key search terms that were used were "rural population, "attitude to health," "health behavior," "health promotion," "health belief," and "health values." Because this was a qualitative review, multiple online databases were searched: PubMed, CINAHL, PsycINFO, AnthroSource, and Sociological Abstracts, followed by a manual search of the reference sections of all included studies. Additional literature was identified using the "see all related articles" function in PubMed and a similar search tool (JANE) that matches abstracts to related articles.

Operational criteria for rural definition of health and comparison groups:

1. Does the article present views, assertions, or findings re: a distinctive rural definition of health?
    a. "Yes" response
        i. Authors present findings or conclusions based on their own research that pertain to "rural definition of health," or
        ii. Authors present findings or conclusions based on their own research that pertain to health behaviors, beliefs or attitudes that reflect a "rural definition of health."

b. "Somewhat" response

    i. Authors present findings or conclusions that pertain to "rural definition of health," drawing primarily on other research or other literature, or
    ii. Authors present findings or conclusions that pertain to health behaviors, beliefs or attitudes that reflect a "rural definition of health," drawing primarily on other research or other literature.

c. "No" response

    i. Fails to meet either "a" or "b" above;
    ii. Article constitutes commentary or a summary of other work; or
    iii. Article pertains primarily to rural health status or rural healthcare.

2. Are rural views or findings compared with another population?

    a. "Yes" if comparison is made to a non-rural population (e.g., urban), with findings of similarities or differences;
    b. "Somewhat" if findings for a rural population are compared to either
        i. Findings for a similar non-rural population from another published study;
        ii. Findings from other populations that are "somewhat" more rural or urban (e.g., comparisons between rural communities of varying degrees of remoteness).
    c. "No" if no comparison is presented, or if comparisons are made using variables other than rural – non-rural (e.g., older vs. younger, male vs. female, white vs. black).

Study selection: The first review of articles determined whether an article pertained to a rural definition of health and if a comparison group was utilized. Reviewers further noted how rural was defined (if defined). All information was captured in a spreadsheet (Microsoft Excel 2010) and analyzed based on rural definition of health (yes [y], no [n] , and somewhat [s]); and whether there was a comparison group (yes [y], no/missing [n] , and somewhat [s]). Two reviewers were randomly assigned to each of the 125 articles and blinded to the other reviewer's comments. With two reviewers and three possible decisions regarding each article, there were nine potential agreement results for the rural definition of health determination, three of which indicated consensus (y/y, n/n and s/s). A first review of the articles resulted in consensus for 59% of the articles (n=74) on rural definition of health. For discordant first reviews, two blinded reviewers not assigned to the original review performed a third review. After the second review, there was consensus on 79% (n=99) for rural definition of health, with 31 articles being identified. The 26 articles on which there were disagreement underwent a third review and three more rural definition of health articles were identified, resulting in 34 articles that included a rural definition of health. Assessment of the existence of a comparison group was conducted in concert with the rural definition of health assessment.

Articles were also classified based on level of evidence, grading from A (highest level of evidence) to C (lowest level of evidence). The vast majority presented uncontrolled data, often qualitative. These articles qualified for a low level of evidence "C5. Supportive evidence from poorly controlled or uncontrolled studies." Other articles provided somewhat stronger evidence, in the "B3. Supportive Evidence from well-conducted cohort studies" or "B4. Supportive evidence from well-conducted case-control studies" range. No study qualified for the highest level of evidence (in the A range).
